# Supplementary material for: Identifying and Characterizing Candidate Genes Contributing to a Grain Yield QTL in Wheat
Source: Plants (Basel). 2023 Dec 20;13(1):26. doi: 10.3390/plants13010026 (PMC10780351; doi:10.3390/plants13010026)
Supplement: Supplementary file 1 [file plants-13-00026-s001.zip › Supplementary figures.pdf]

a)

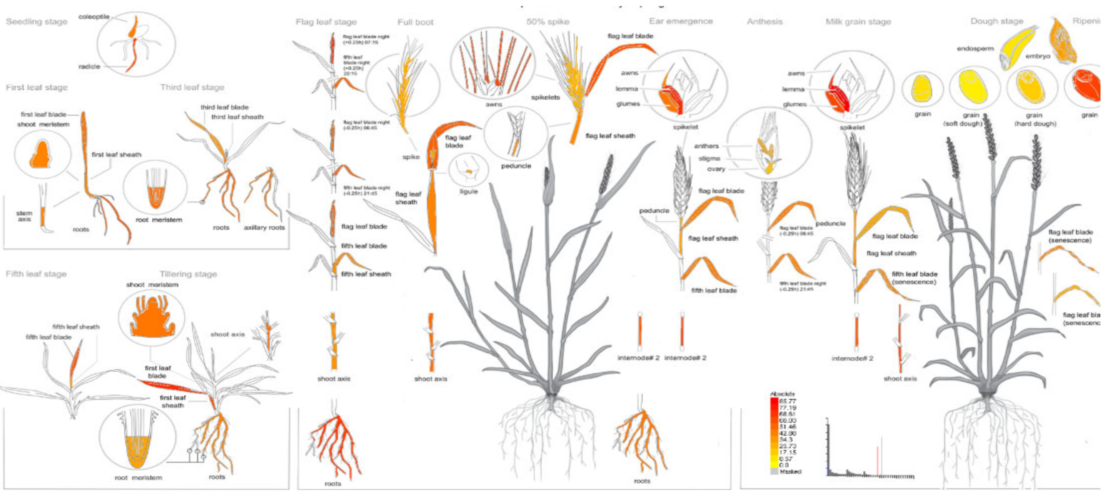
Seedling stage
coleoptile
radicle
First leaf stage
first leaf blade
shoot meristem
first leaf sheath
stem axis
roots
root meristem
Third leaf stage
third leaf blade
third leaf sheath
axillary roots
Fifth leaf stage
Tillering stage
shoot meristem
fifth leaf sheath
fifth leaf blade
first leaf blade
first leaf sheath
shoot axis
root meristem
Flag leaf stage
Full boot
50% spike
Ear emergence
Anthesis
Milk grain stage
Dough stage
flag leaf blade
fifth leaf blade
fifth leaf sheath
spike
flag leaf sheath
awns
spikelets
ligule
peduncle
lemma
glumes
spikelet
anthers
stigma
ovary
internode# 2
endosperm
embryo
grain
grain (soft dough)
grain (hard dough)
flag leaf blade (senescence)
fifth leaf blade (senescence)
Masked

b)

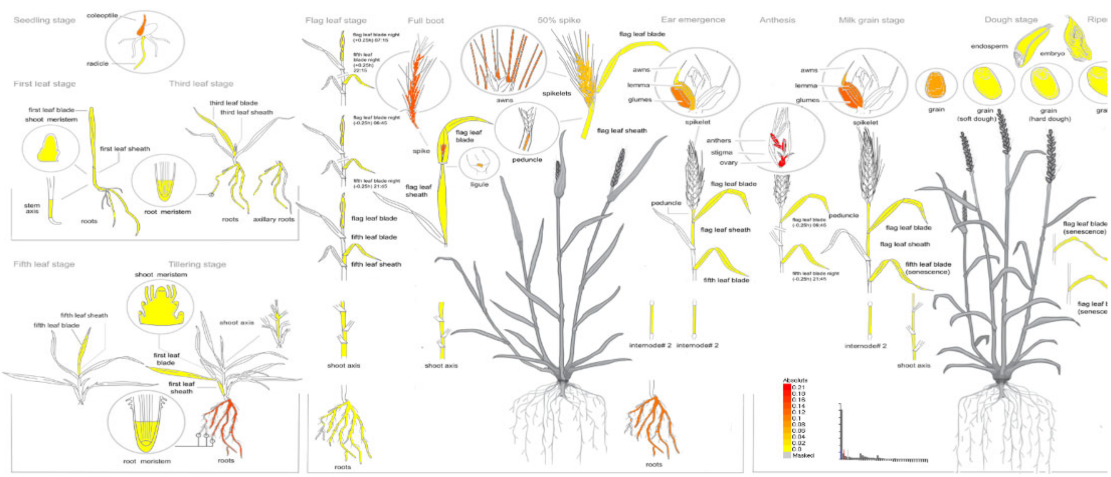
Seedling stage
coleoptile
radicle
First leaf stage
first leaf blade
shoot meristem
first leaf sheath
stem axis
roots
root meristem
Third leaf stage
third leaf blade
third leaf sheath
axillary roots
Fifth leaf stage
Tillering stage
shoot meristem
fifth leaf sheath
fifth leaf blade
first leaf blade
first leaf sheath
shoot axis
root meristem
Flag leaf stage
Full boot
50% spike
Ear emergence
Anthesis
Milk grain stage
Dough stage
flag leaf blade
fifth leaf blade
fifth leaf sheath
spike
flag leaf sheath
awns
spikelets
ligule
peduncle
lemma
glumes
spikelet
anthers
stigma
ovary
internode# 2
endosperm
embryo
grain
grain (soft dough)
grain (hard dough)
flag leaf blade (senescence)
fifth leaf blade (senescence)
Masked

c)

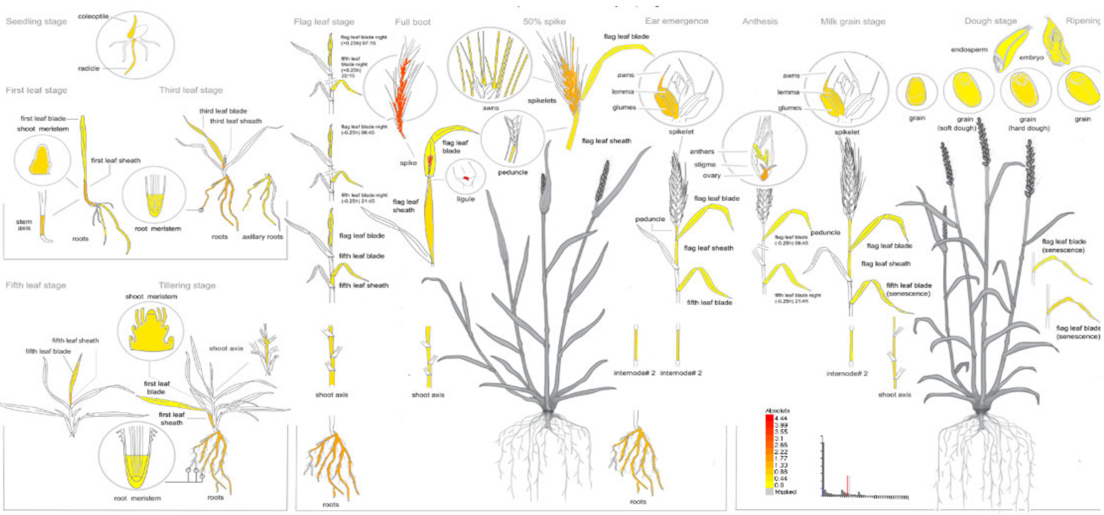
Seedling stage
coleoptile
radicle
First leaf stage
first leaf blade
shoot meristem
first leaf sheath
stem axis
roots
root meristem
Third leaf stage
third leaf blade
third leaf sheath
axillary roots
Fifth leaf stage
Tillering stage
shoot meristem
fifth leaf sheath
fifth leaf blade
first leaf blade
first leaf sheath
shoot axis
root meristem
Flag leaf stage
Full boot
50% spike
Ear emergence
Anthesis
Milk grain stage
Dough stage
Ripening
flag leaf blade
fifth leaf blade
fifth leaf sheath
spike
flag leaf sheath
awns
spikelets
ligule
peduncle
lemma
glumes
spikelet
anthers
stigma
ovary
internode# 2
endosperm
embryo
grain
grain (soft dough)
grain (hard dough)
flag leaf blade (senescence)
fifth leaf blade (senescence)
Masked

d)

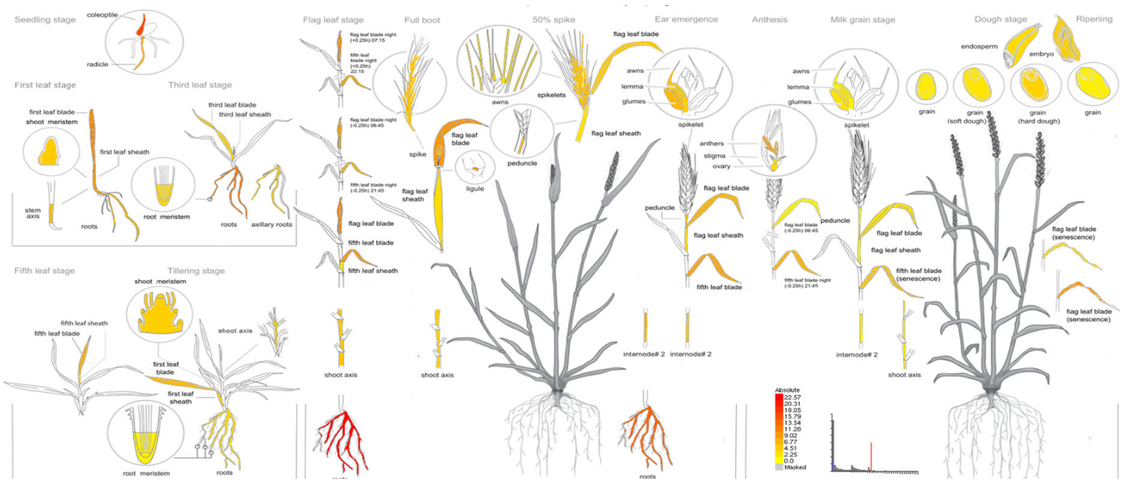

Figure S1: Expression of the candidate genes in different parts of the wheat plant. The level of gene expression is indicated by the colour chart present at the lower right side of the picture. a) Sulfite reductase gene, b) Glutamine synthetase gene, c) *Ferredoxin*-like gene, and d) *Tetratricopetide* protein gene.

a) TraesCS1B02G336300

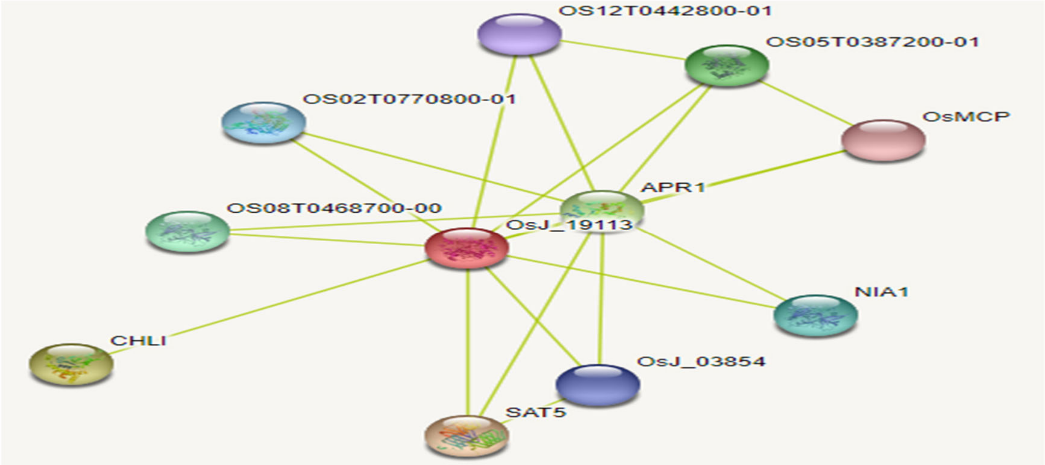

OsMCP = Moco containing protein, APR1 = Adenylylsulfate reductase 1, CHL1 = Magnesium-chelatase subunit ChlI, SAT5 = Serine acetyltransferase 5, and NIA1 = Nitrate reductase apoenzyme 1

b) TraesCS1B02G336800

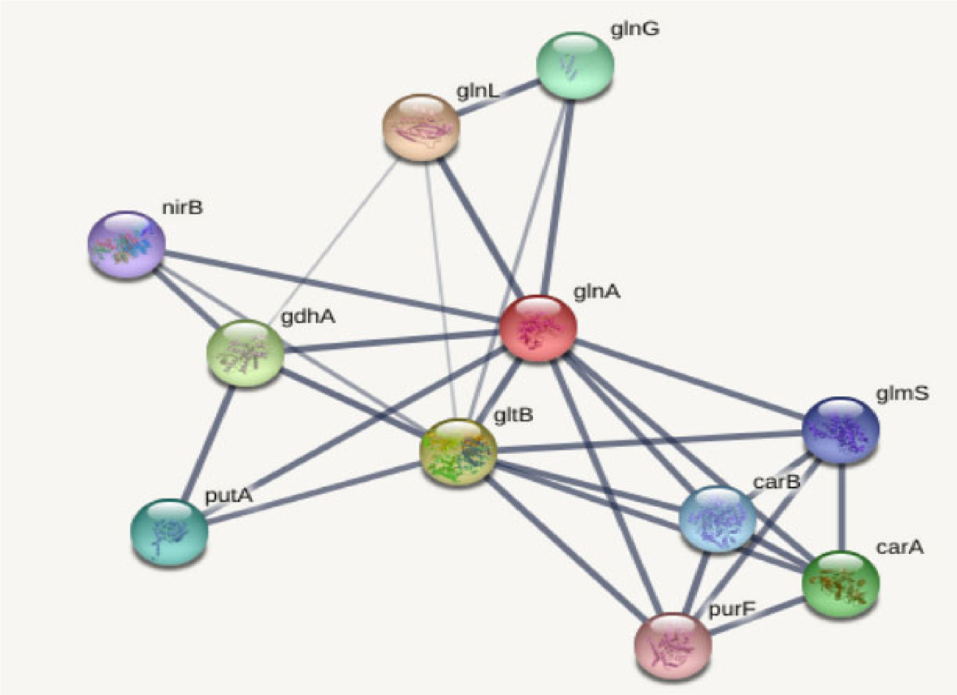

nirB: involved in anaerobic respiration; gdhA: Catalyzes the reversible oxidative deamination of glutamate; PutA: Oxidizes proline to glutamate; NtrC & NtrB: controls expression of the nitrogen-regulated (ntr) genes; gltB: Catalyzes the conversion of L-glutamine and 2- oxoglutarate; purF: Catalyzes the formation of phosphoribosylamine; carA & carB: involved in arginine biosynthetic and pyrimidine nucleotide biosynthetic process; glmS: Catalyzes the hexosamine metabolism

c) TraesCS1B02G337100

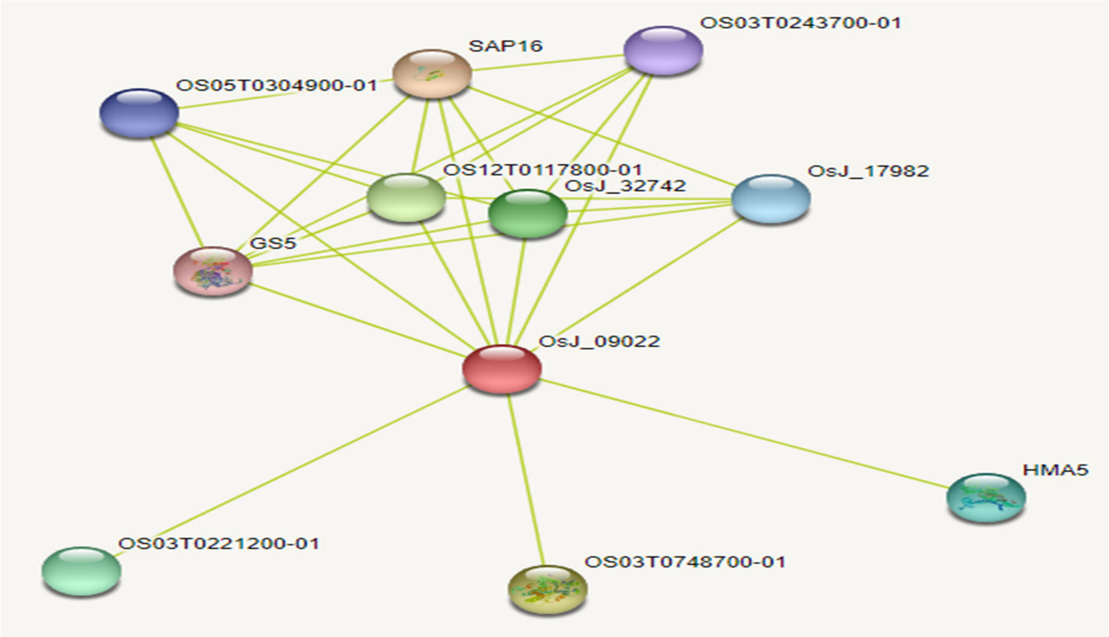

GS5 = grain size 5 gene, HAM5 = gene involved in loading Cu; SAP16 = stress associated protein gene 16.

d) TraesCS1B02G337400

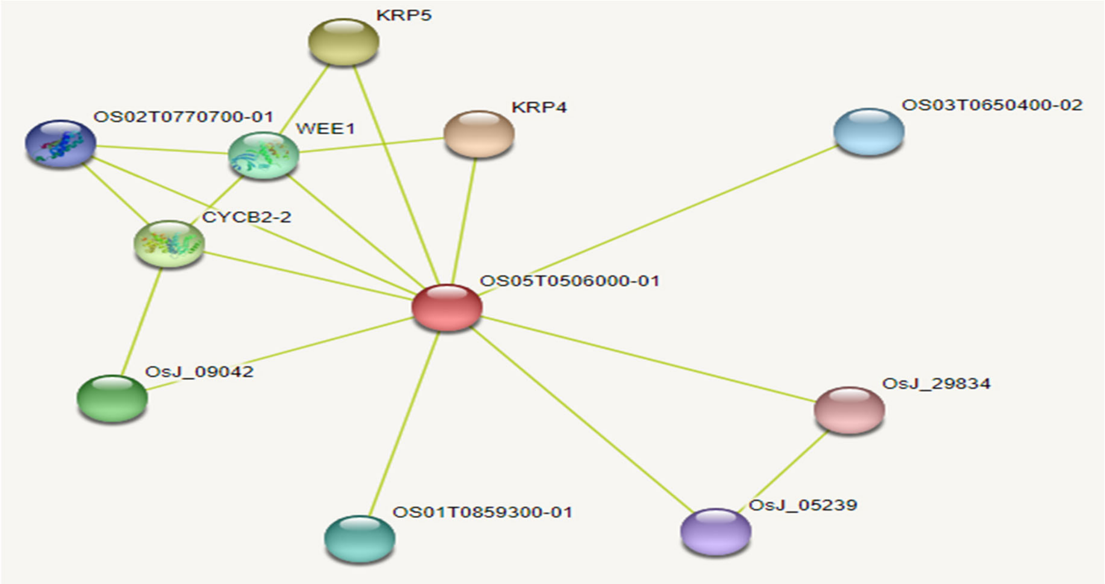

KRP5 = KIP-related protein 5, WEE1 = wall-associated receptor-like protein kinase; KRP4 = KIP-related protein 4, and CYCB2-2 = G2/mitotic-specific cyclin-B2-2.

Figure S2: Protein-protein interaction of selected genes obtained from the STRING database

a) Involvement of sulphite reductase and *ferredoxin* gene in the metabolic pathway showing synthesis of sulpfur containing amino acids

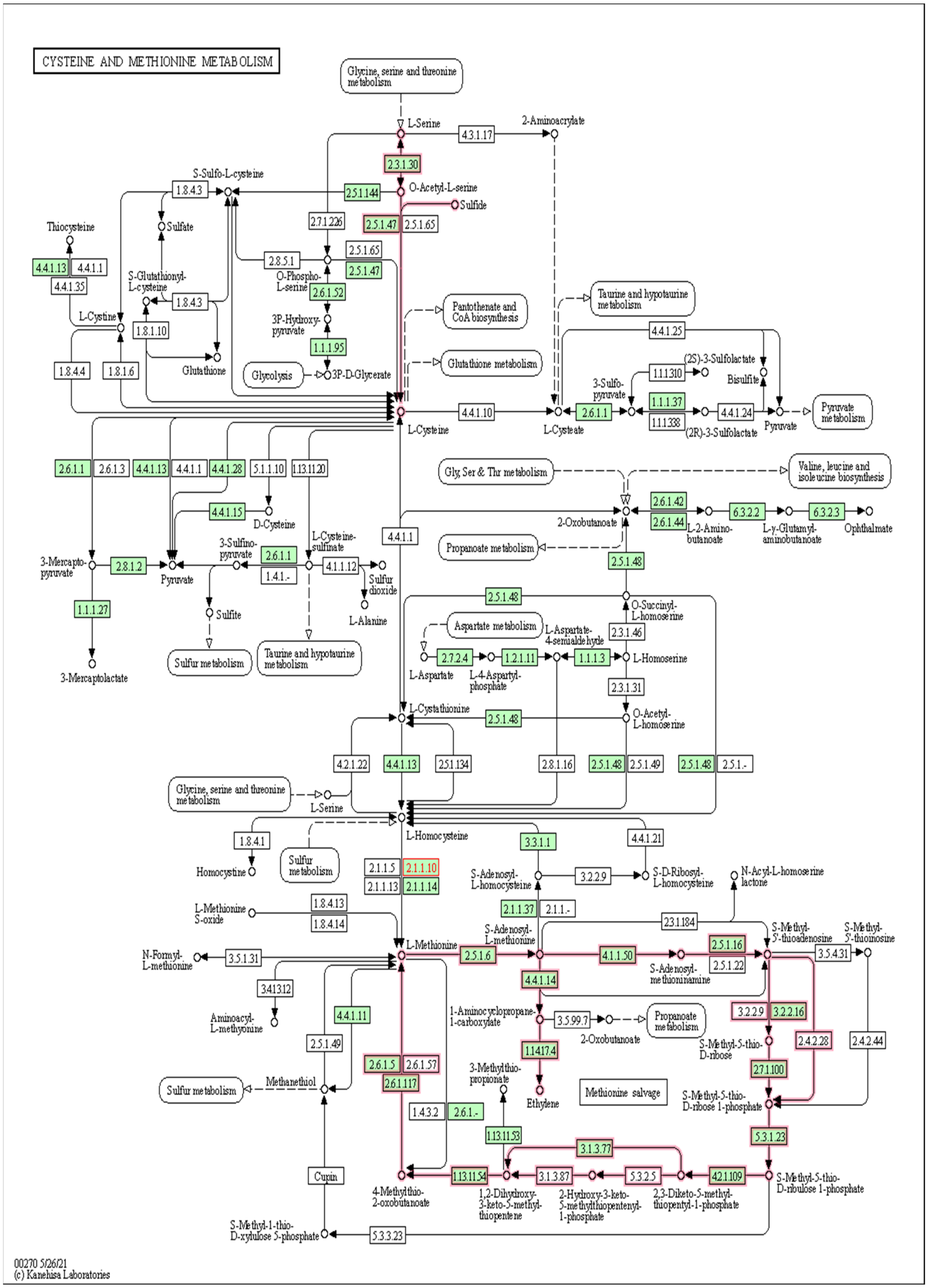

b) Involvement of *ferredoxin* gene in the MAPK signaling pathway which in turn regulate transcription

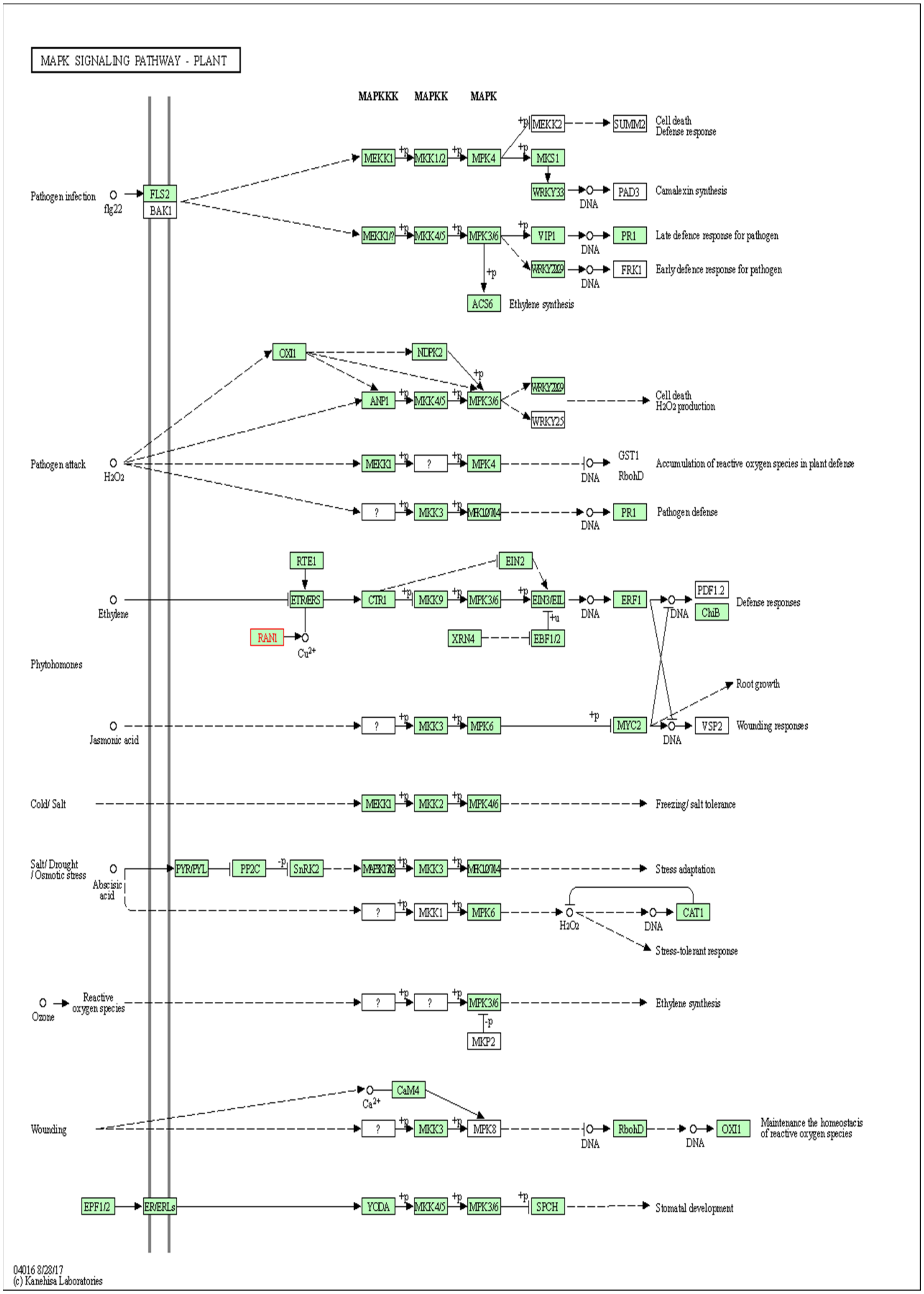

c) Involvement of sulphite reductase and *tetratricopetide*-repeat gene in sulpfur metabolism

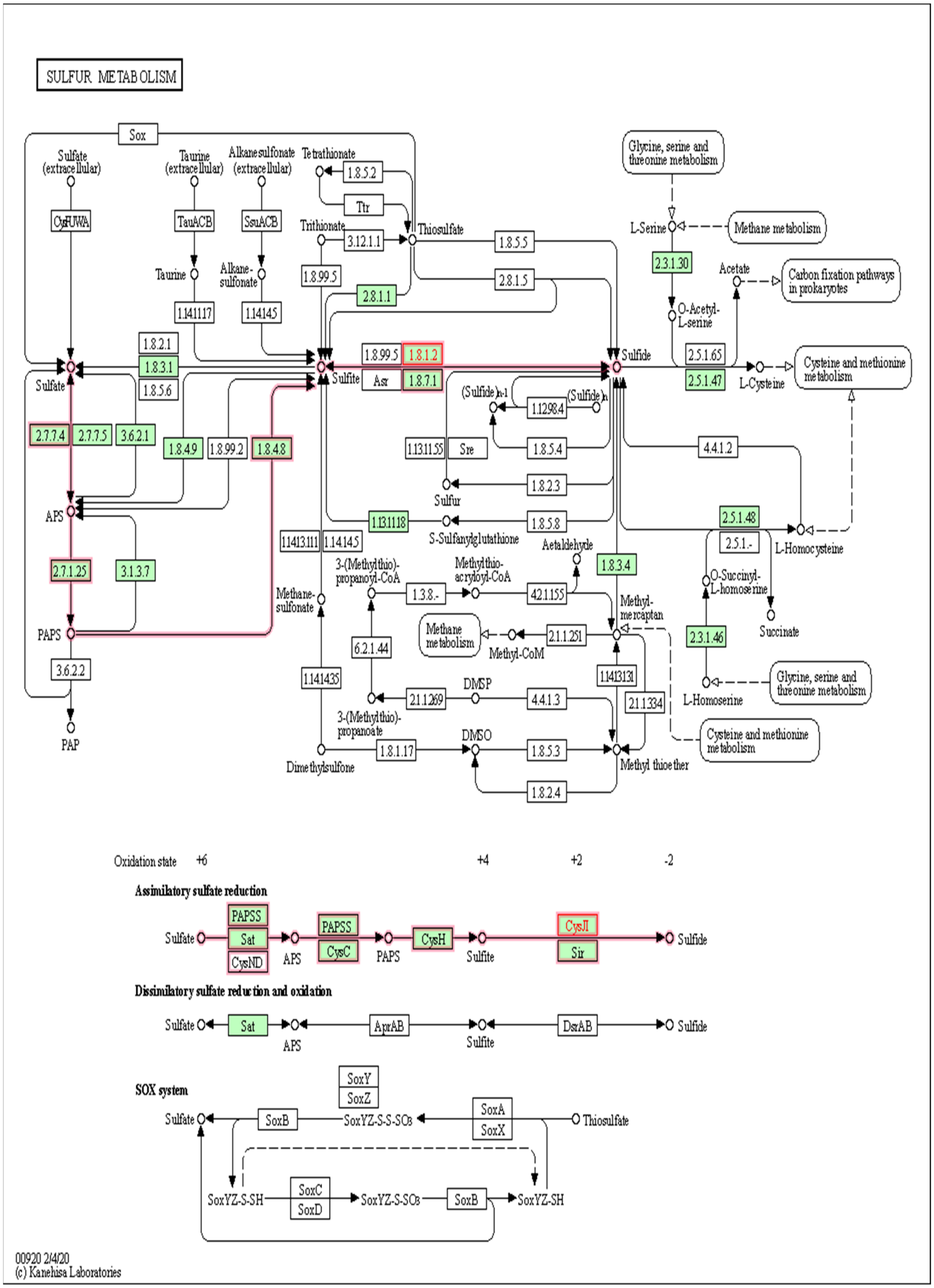

d) Involvement of sulphite reductase gene in the metabolic pathway showing sulpfur relay system

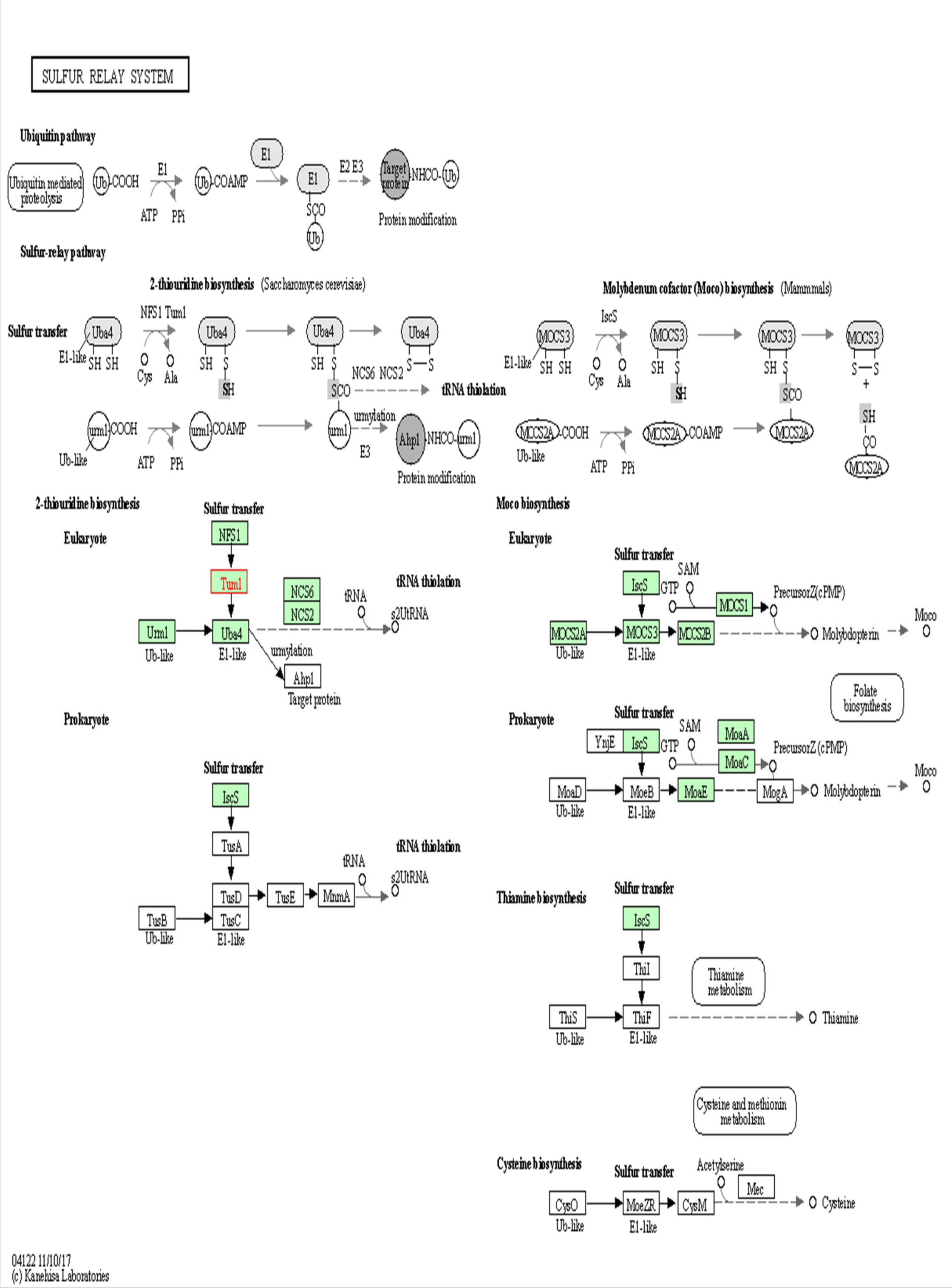

e) Involvement of sulphite reductase and glutamine synthetase gene in alanine, aspartate and glutamine metabolism

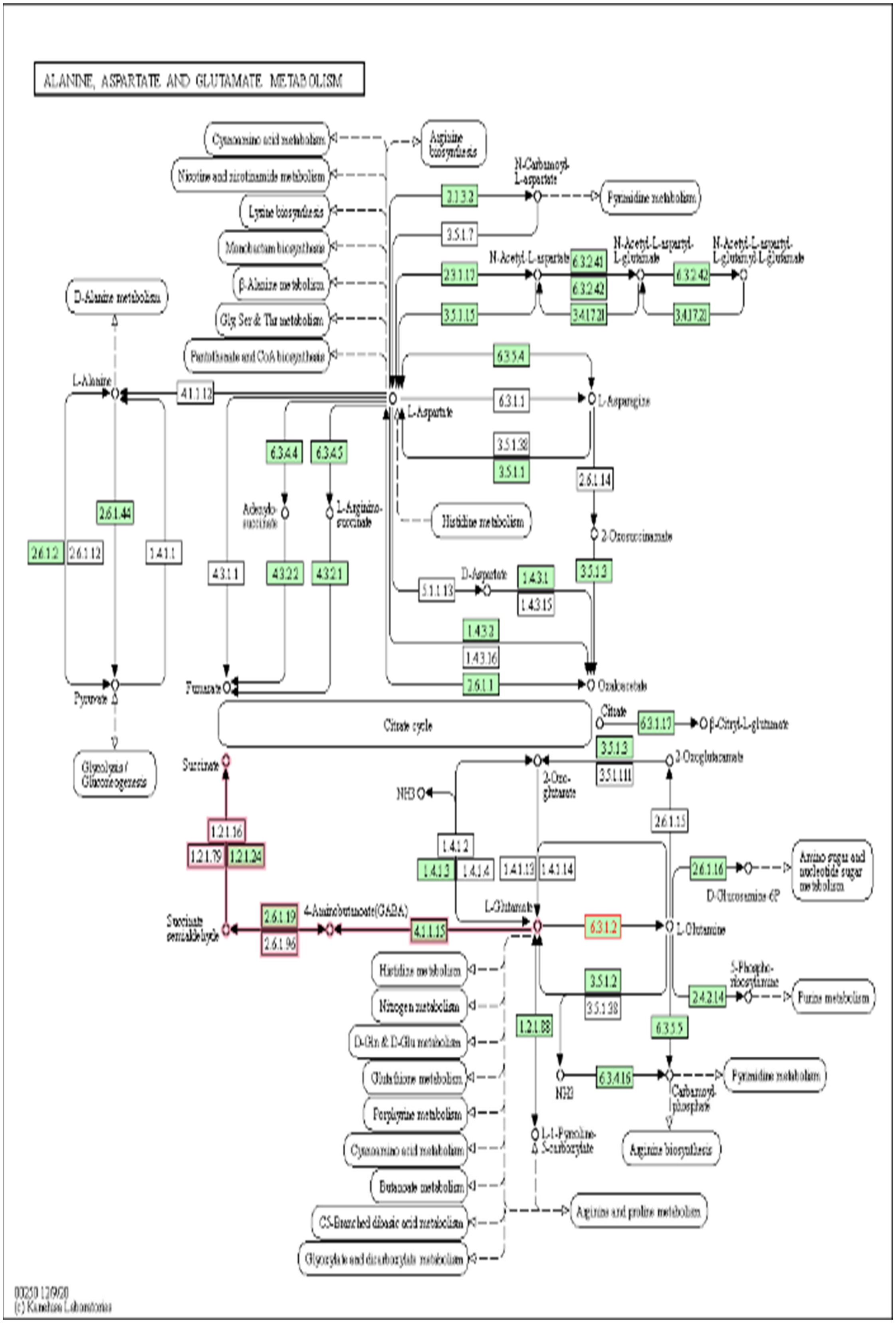

f) Involvement of *tetratricopetide*-repeat gene in the plant hormone signal transduction

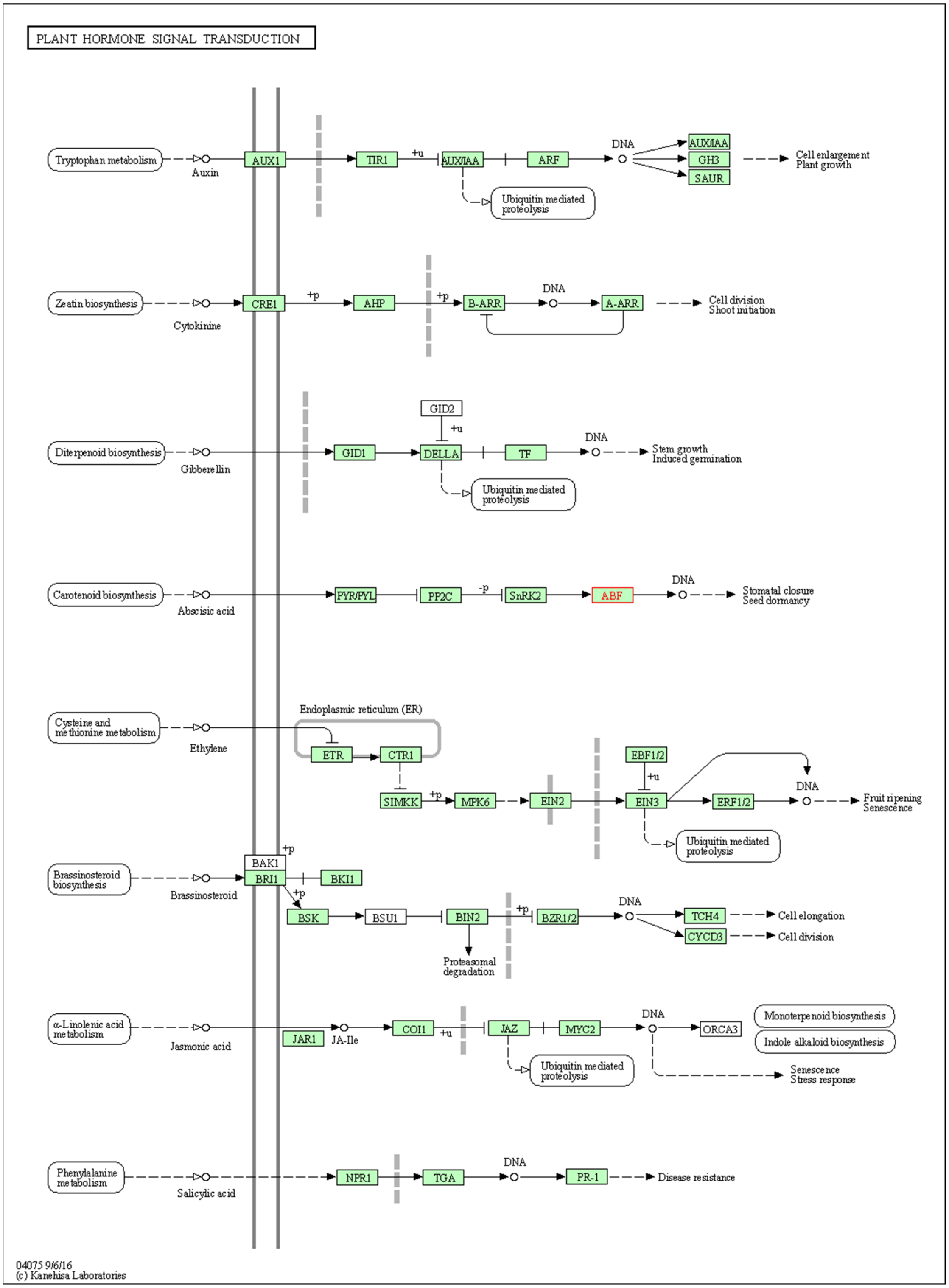

g) Involvement of glutamine synthetase gene in nitrogen metabolism

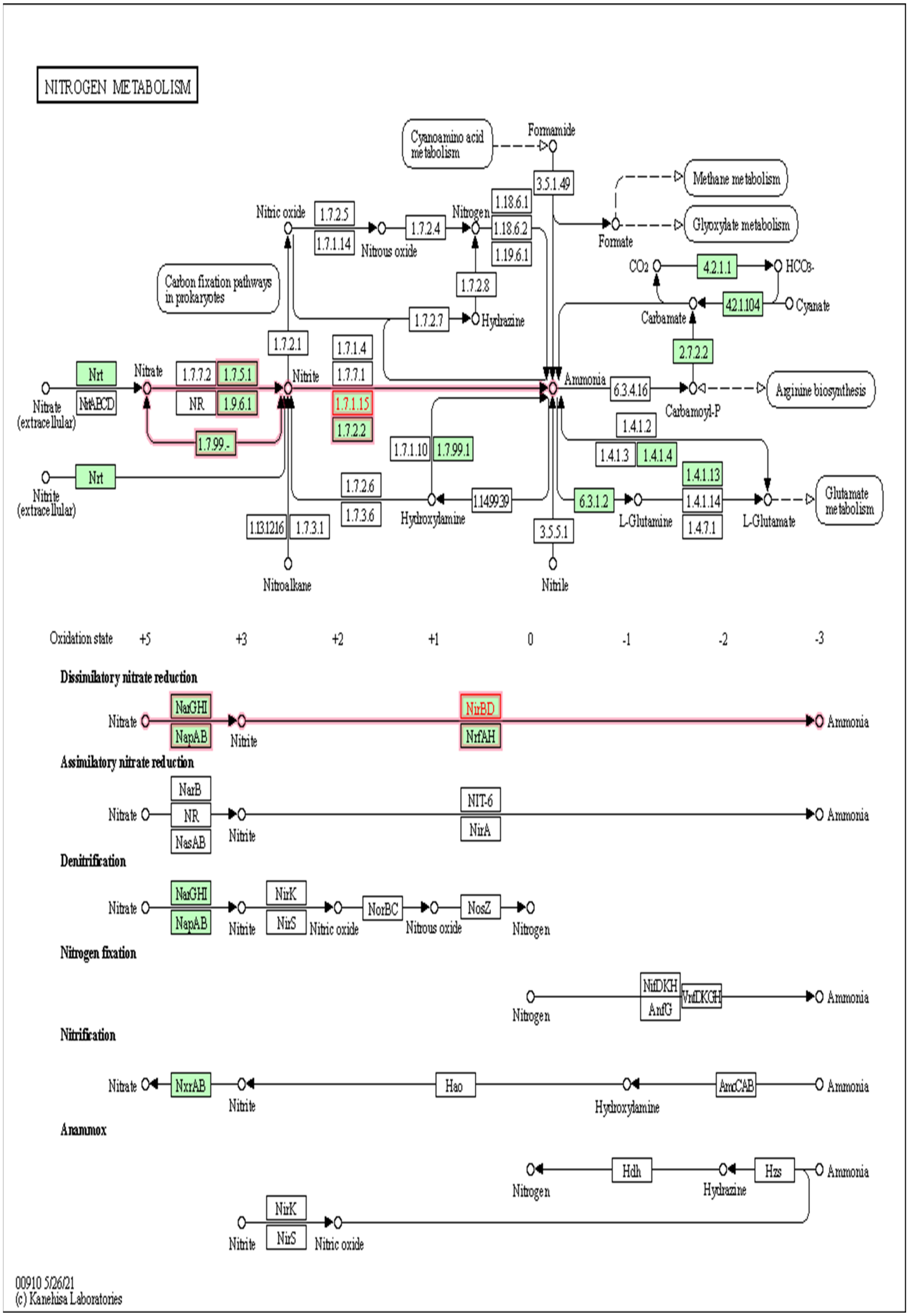

h) Involvement of glutamine synthase gene in arginine and proline metabolism

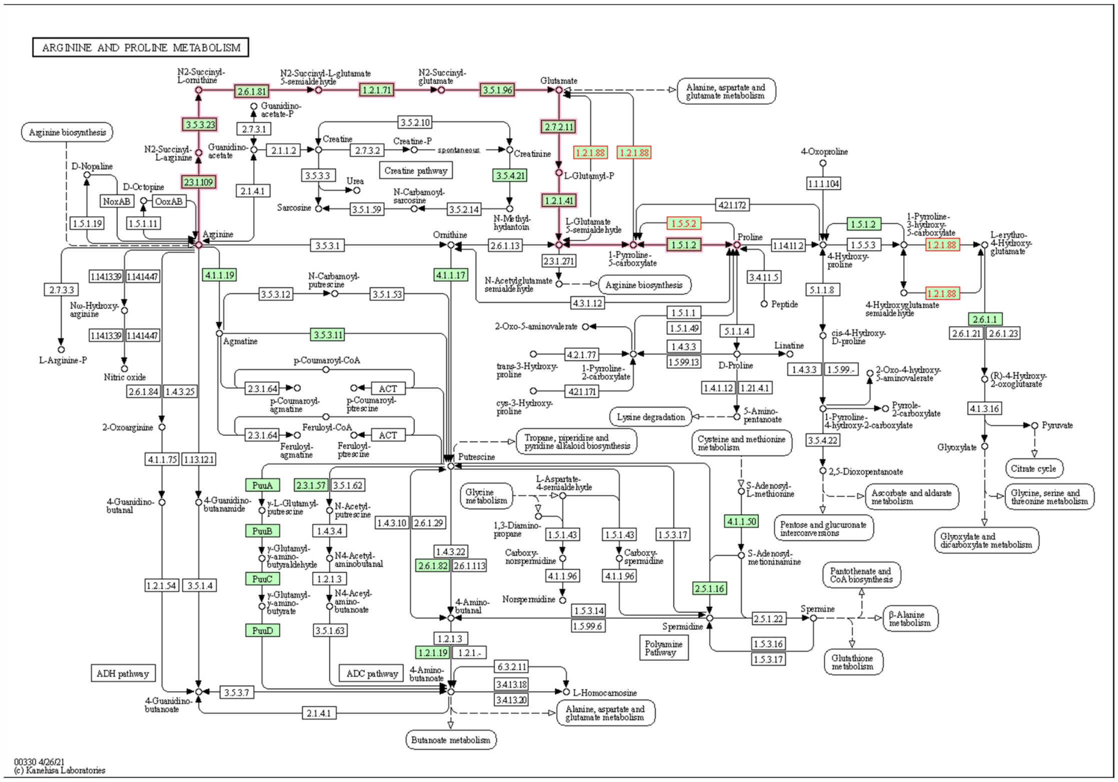

i) Involvement of glutamine synthase gene in arginine biosynthesis

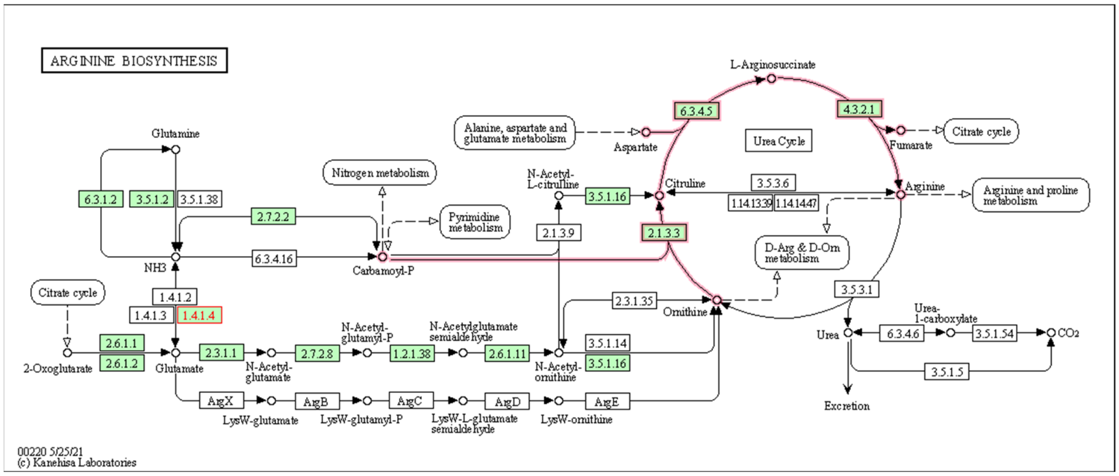

j) Involvement of glutamine synthase gene in amino sugar and nucleic acid sugar metabolism

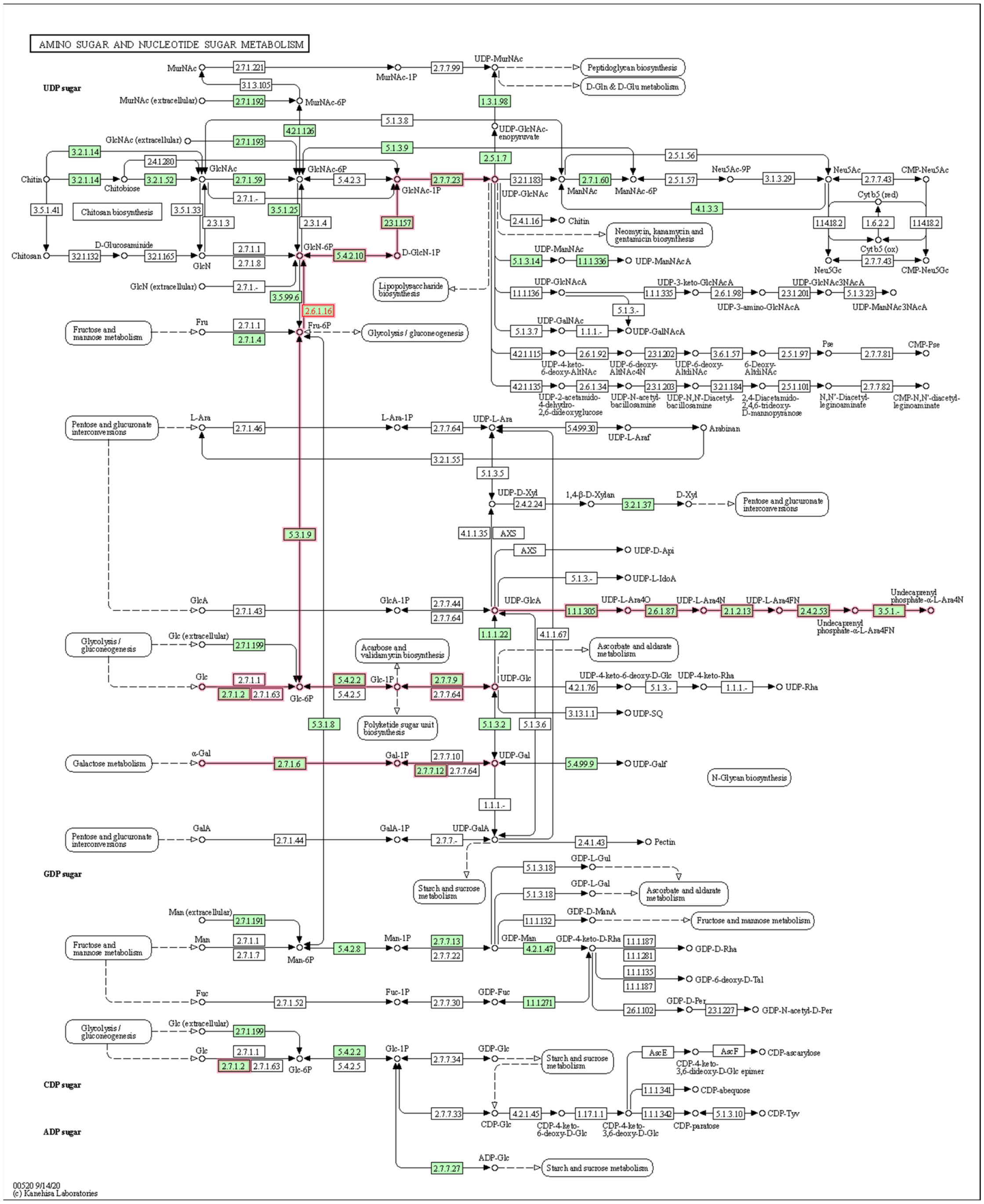

k) Involvement of glutamine synthase gene in pyrimidine metabolism

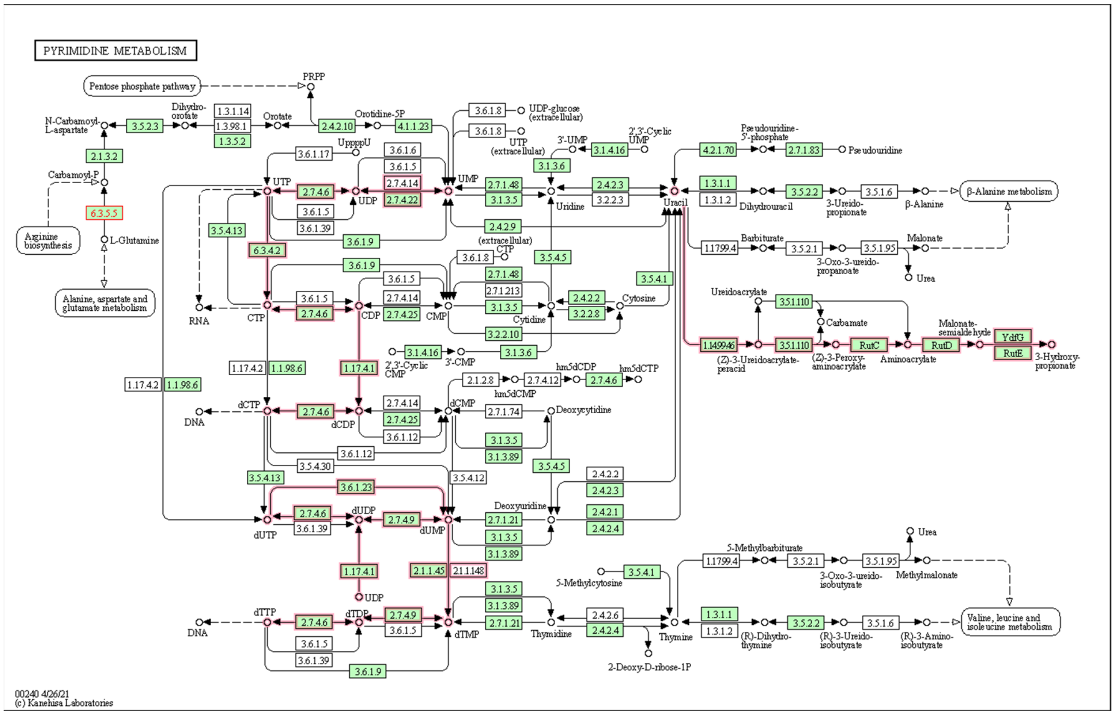

l) Involvement of glutamine synthase gene in glyoxylate, and dicarboxylate metabolism

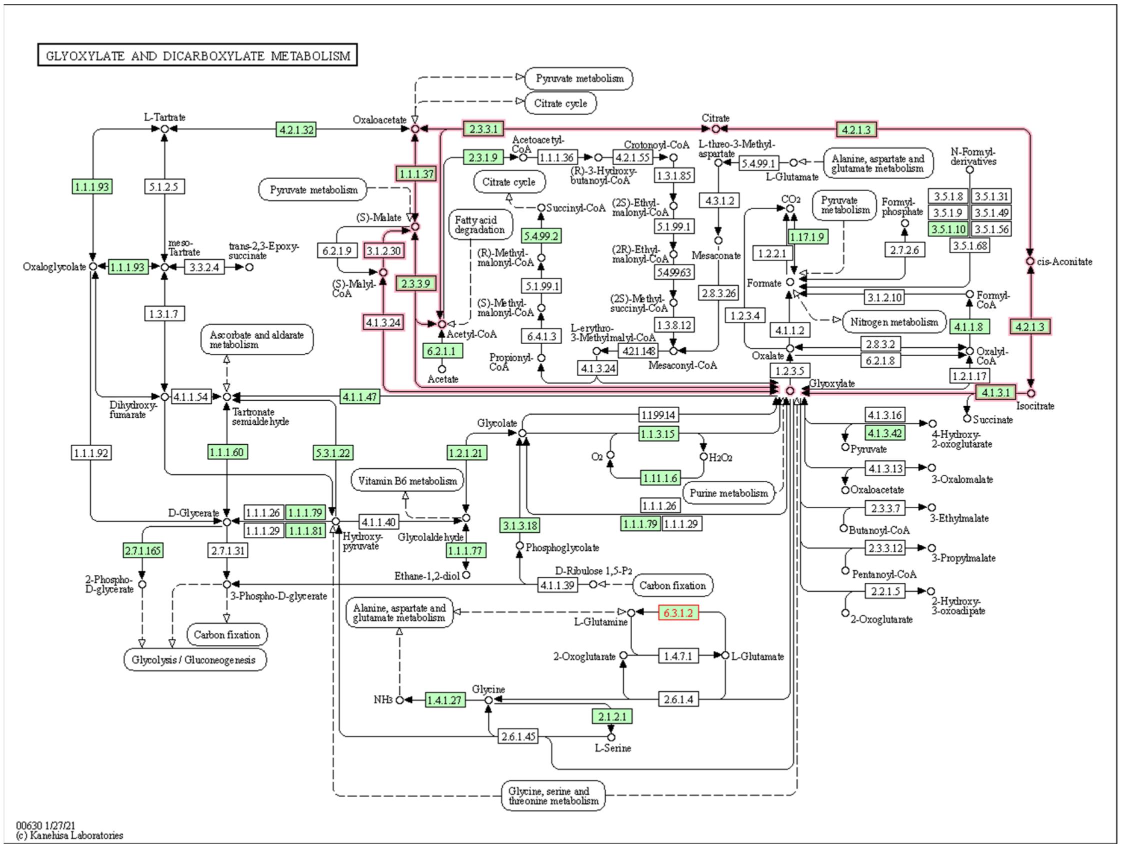

Figure S3: Involvement in KEGG pathway for selected genes

a) Sulphite reductase gene

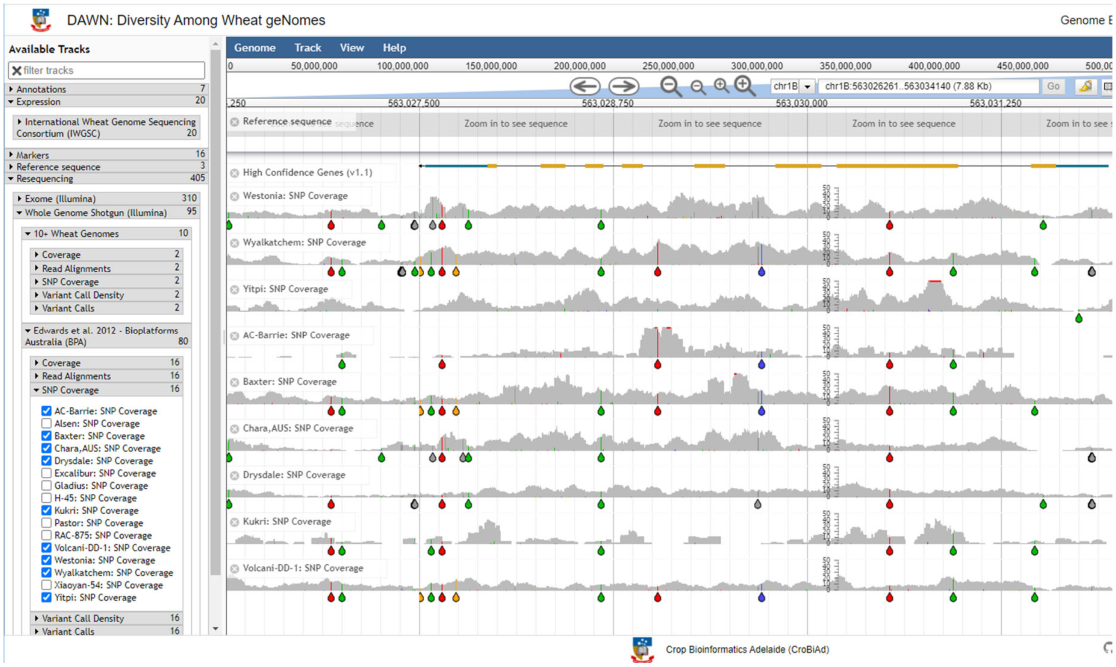

b) Glutamine synthetase gene

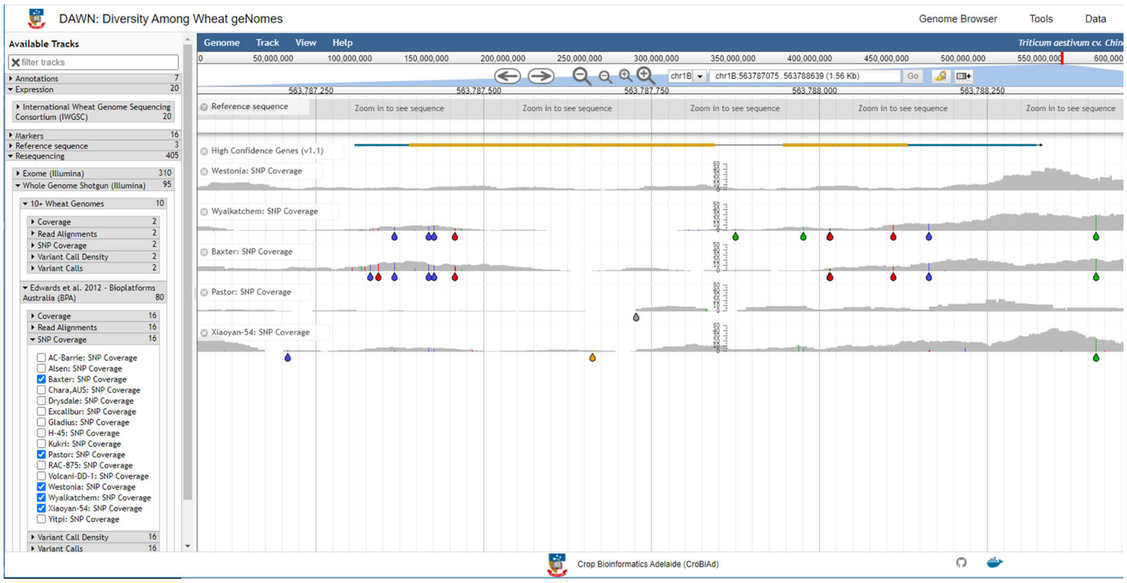

c) *Ferredoxin* gene

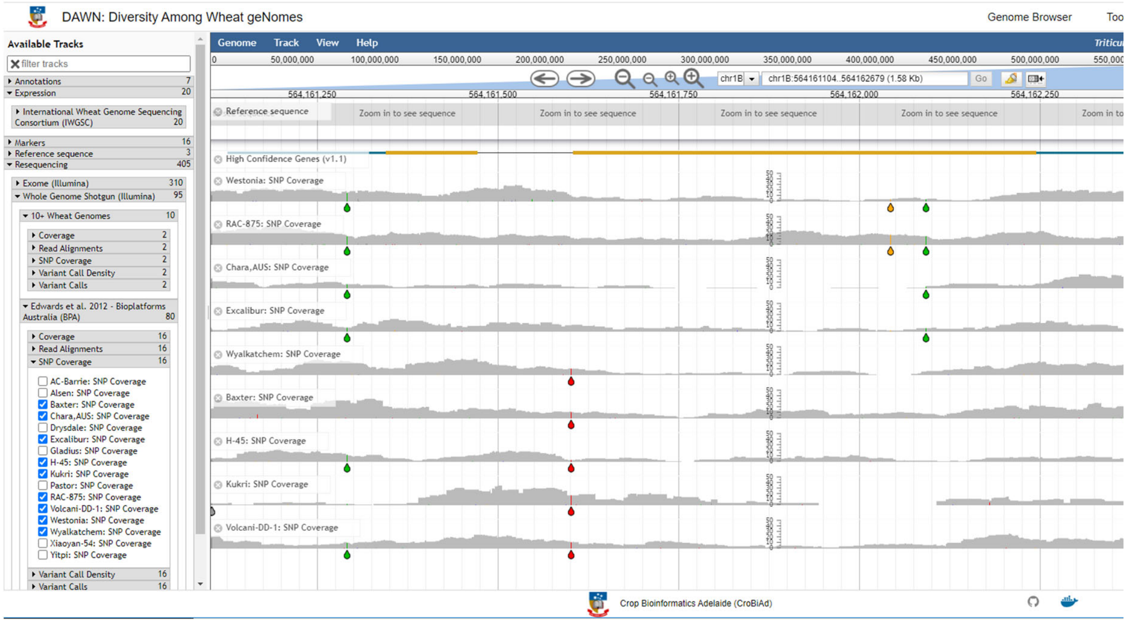

d) *Tetratricopetide*-repeat gene

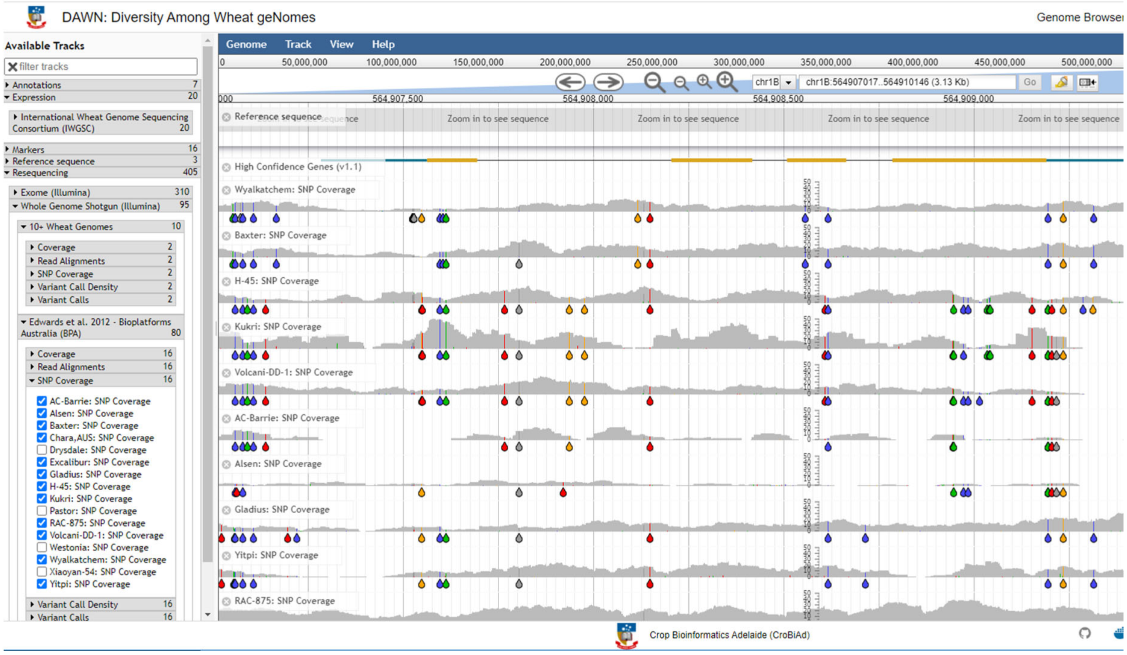

Figure S4: Variation in DNA sequence obtained from DAWN database (Haigh *et al.*, 2018).

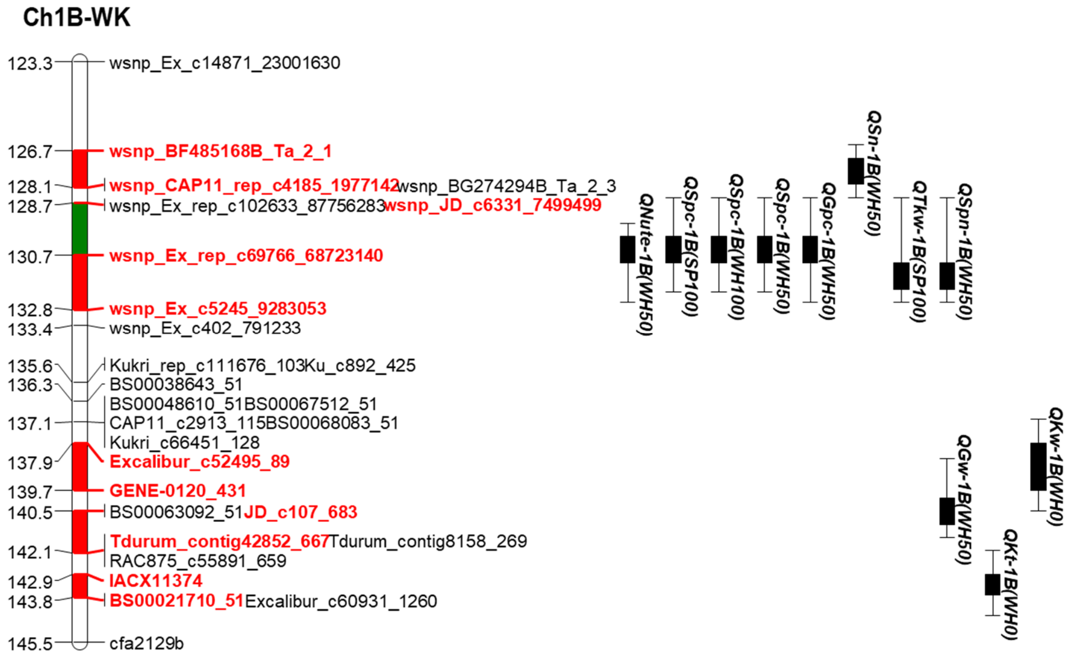

Figure S5: Marker position for different QTLs at chromosome 1B.
