# Supplementary material for: Identifying and Characterizing Candidate Genes Contributing to a Grain Yield QTL in Wheat
Source: Plants (Basel). 2023 Dec 20;13(1):26. doi: 10.3390/plants13010026 (PMC10780351; doi:10.3390/plants13010026)
Supplement: Supplementary file 1 [file plants-13-00026-s001.zip › Supplementary tables.pdf]

Table S1: QTLs detected on chromosome 1BL in wheat DH population Westonia and Kauz

Trait Name	Env.	Position	Left Marker	Right Marker	LOD	PVE (%)	Add
Seed number	wh50	128	NM515	NM854	3.91	8.61	-2.57
Straw protein content	sh100	130	wsnp_JD_c6331_7499499	wsnp_Ex_rep_c69766_68723140	4.62	10.18	0.13
Straw protein content	wh100	130	wsnp_JD_c6331_7499499	wsnp_Ex_rep_c69766_68723140	3.53	12.74	0.13
Straw protein content	wh50	131	wsnp_Ex_rep_c69766_68723140	wsnp_Ex_c5245_9283053	8.68	23.11	0.19
Grain protein content	wh50	131	wsnp_Ex_rep_c69766_68723140	wsnp_Ex_c5245_9283053	2.70	9.81	0.25
N utilization efficiency	wh50	131	wsnp_Ex_rep_c69766_68723140	wsnp_Ex_c5245_9283053	5.02	16.14	-0.003
Thousand kernels' weight	sh100	131	wsnp_Ex_rep_c69766_68723140	wsnp_Ex_c5245_9283053	1.86	3.21	-0.64
Spikelet number	wh50	131	wsnp_Ex_rep_c69766_68723140	wsnp_Ex_c5245_9283053	2.25	6.30	-0.30
Kernel width	wh0	138	E27324	GENE0120_431	1.74	2.86	0.02
Grain weight	wh50	141	JD_c107_683	TC71367	2.01	7.034	-0.33
Kernel thickness	wh0	143	IACX11374	BS00021710_51	2.69	5.72	-0.03

Table S2: Example of information retrieved from the Functional Annotation table for candidate genes

**Feature ID**	TraesCS1A01G002000.1	TraesCS1A01G003100.1
**RefSeq protein ID**	tr|A0A072UPV9|A0A072UPV9_MEDTR	AT5G17680.1
**Annotation description**	RING-finger ubiquitin ligase	disease resistance protein (TIR-NBS-LRR class)
**Description**	PF13639: Ring finger domain	PF00931: NB-ARC domain
**Go annotations**	GO:0005515 MF: protein binding; GO:0008270 MF: zinc ion binding	GO:0043531 MF: ADP binding
**Protein domains**	PF13639	PF00931
**InterPro**	IPR001841; IPR013083	IPR002182; IPR011991; IPR027417; IPR032675
**Ontology**	GO:0005515; GO:0008270	GO:0043531
